# Supplementary material for: PKN1 Exerts Neurodegenerative Effects in an In Vitro Model of Cerebellar Hypoxic–Ischemic Encephalopathy via Inhibition of AKT/GSK3β Signaling
Source: Biomolecules. 2023 Oct 31;13(11):1599. doi: 10.3390/biom13111599 (PMC10669522; doi:10.3390/biom13111599)
Supplement: Supplementary file 1 [file biomolecules-13-01599-s001.zip › biomolecules-2664949-supplementary.pdf]

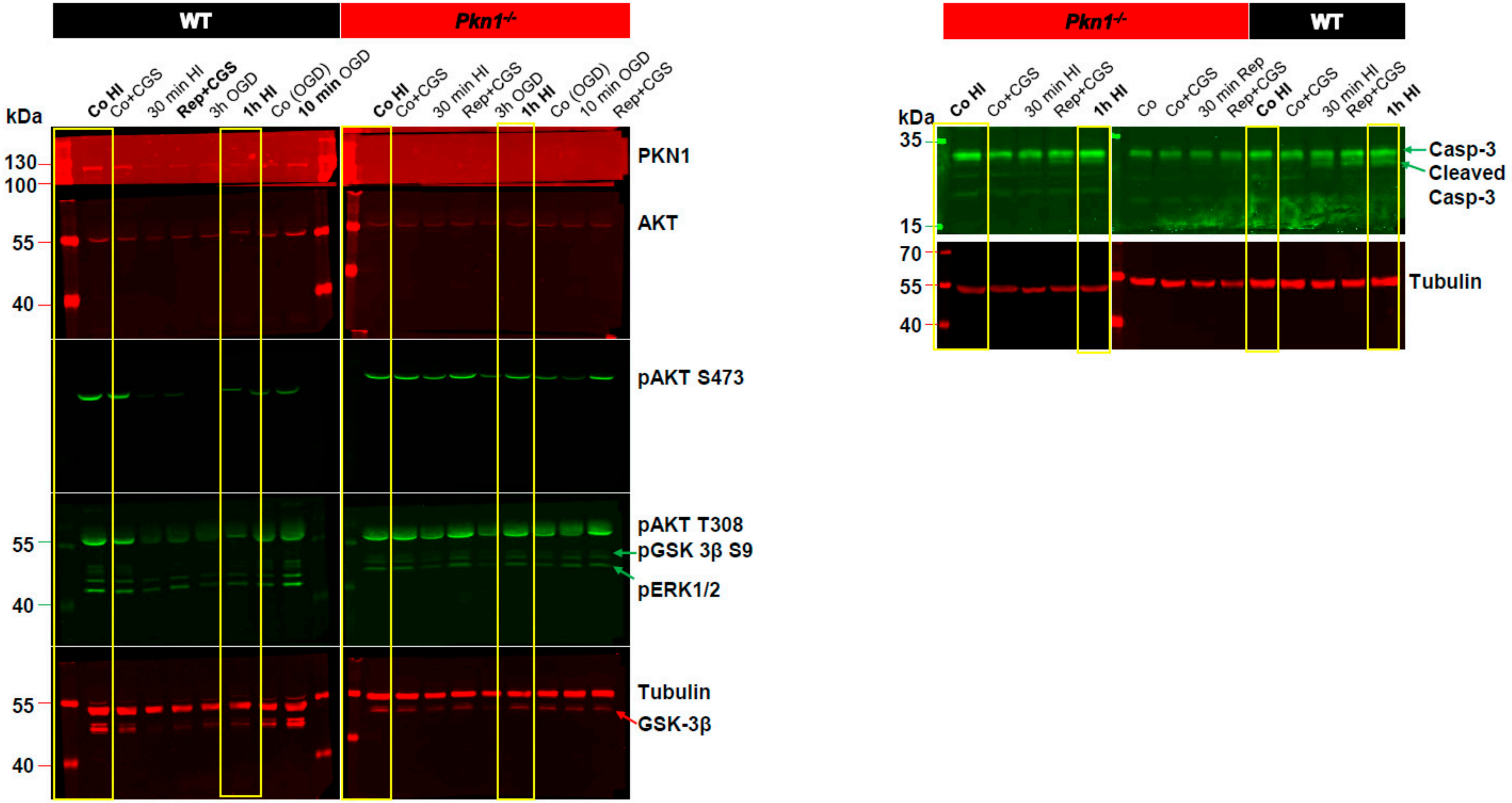

Figure S1. Uncropped blots from Figure 1a. Lanes taken for the show blot are shown in yellow.

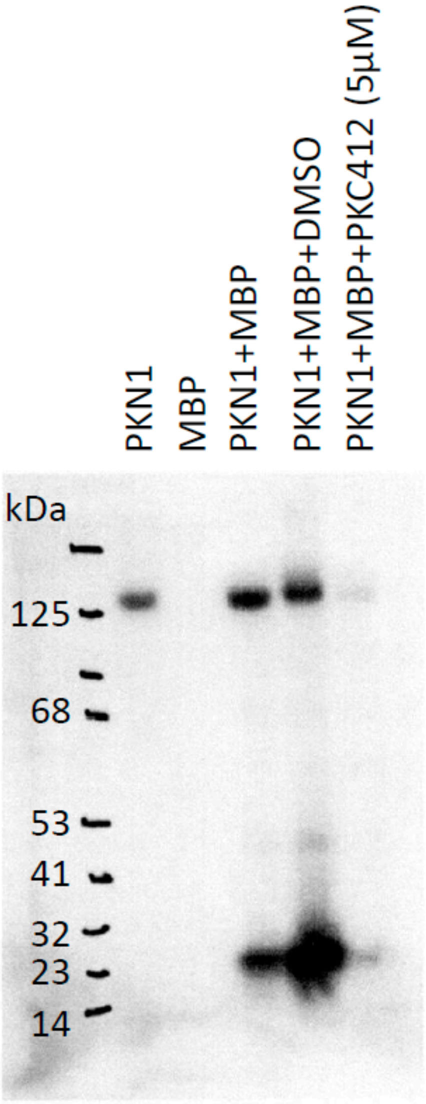

Figure S2. Uncropped Blot from Figure 3a.

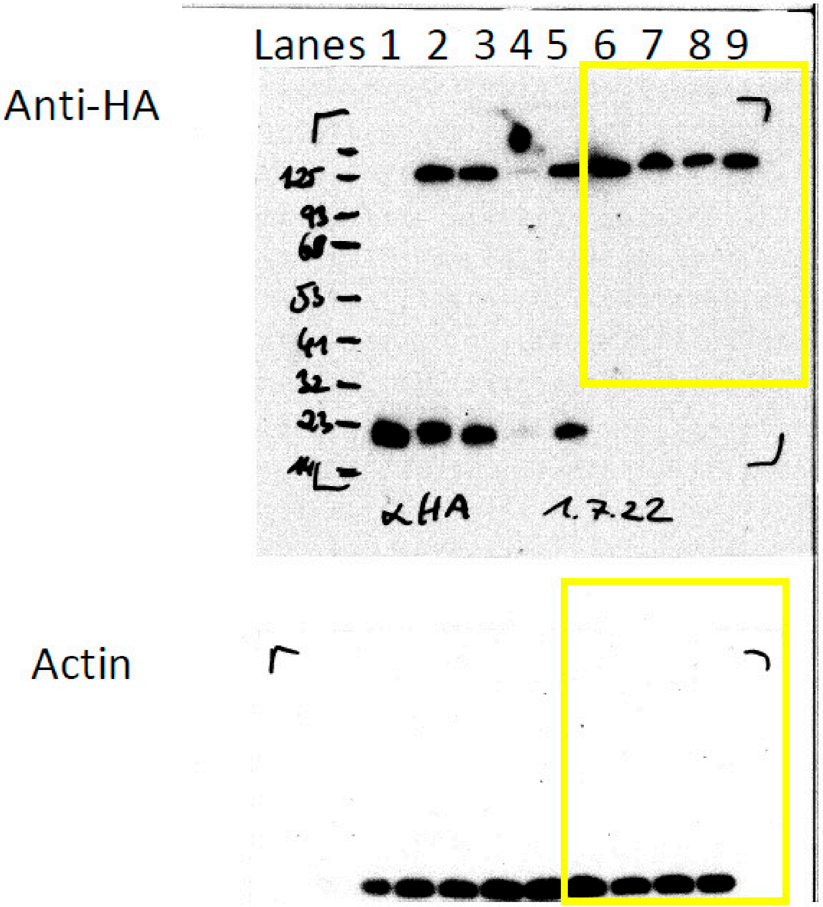

Lanes

1 15 µg RhoAG14V + 15 µg pEFneo
2 15 µg RhoAG14V + 15 µg *h*PKN1 wt
3 15 µg RhoAG14V + 15 µg *h*PKN1 TT63/64AA
4 15 µg RhoAG14V + 15 µg *h*PKN1 S374A
5 15 µg RhoAG14V + 15 µg *h*PKN1 T774A
6 15 µg pEFneo + 15 µg *h*PKN1 wt
7 15 µg pEFneo + 15 µg *h*PKN1 TT63/64AA
8 15 µg pEFneo + 15 µg *h*PKN1 S374A
9 15 µg pEFneo + 15 µg *h*PKN1 T774A

Figure S3. Uncropped blots from Figure 4a. Lanes taken for the show blot are shown in yellow.

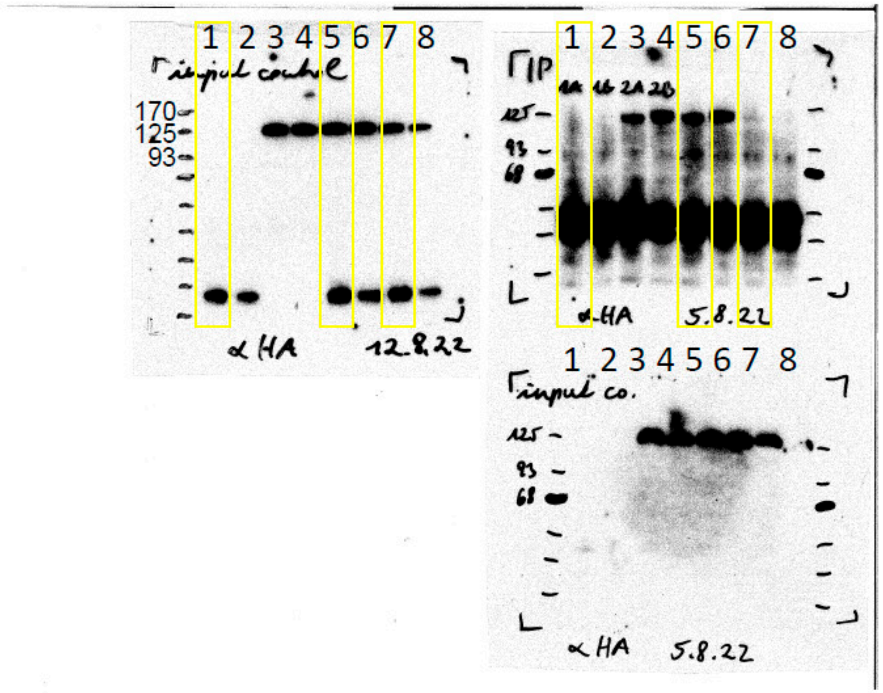

Lanes

1 15 µg RhoAG14V + 15 µg pEFneo (control)

2 15 µg RhoAG14V + 15 µg pEFneo (PdBu/Ionomycin)

3 15 µg pEFneo + 15 µg *h*PKN1 wt(control)

4 15 µg pEFneo + 15 µg *h*PKN1 wt(PdBu/Ionomycin)

5 15 µg RhoAG14V + 15 µg *h*PKN1 wt(control)

6 15 µg RhoAG14V + 15 µg *h*PKN1 wt(PdBu/Ionomycin)

7 15 µg RhoAG14V + 15 µg *h*PKN1 S374A(control)

8 15 µg RhoAG14V + 15 µg *h*PKN1 S374A(PdBu/Ionomycin)

Figure S4. Uncropped blots from Figure 5a. Lanes taken for the show blot are shown in yellow.

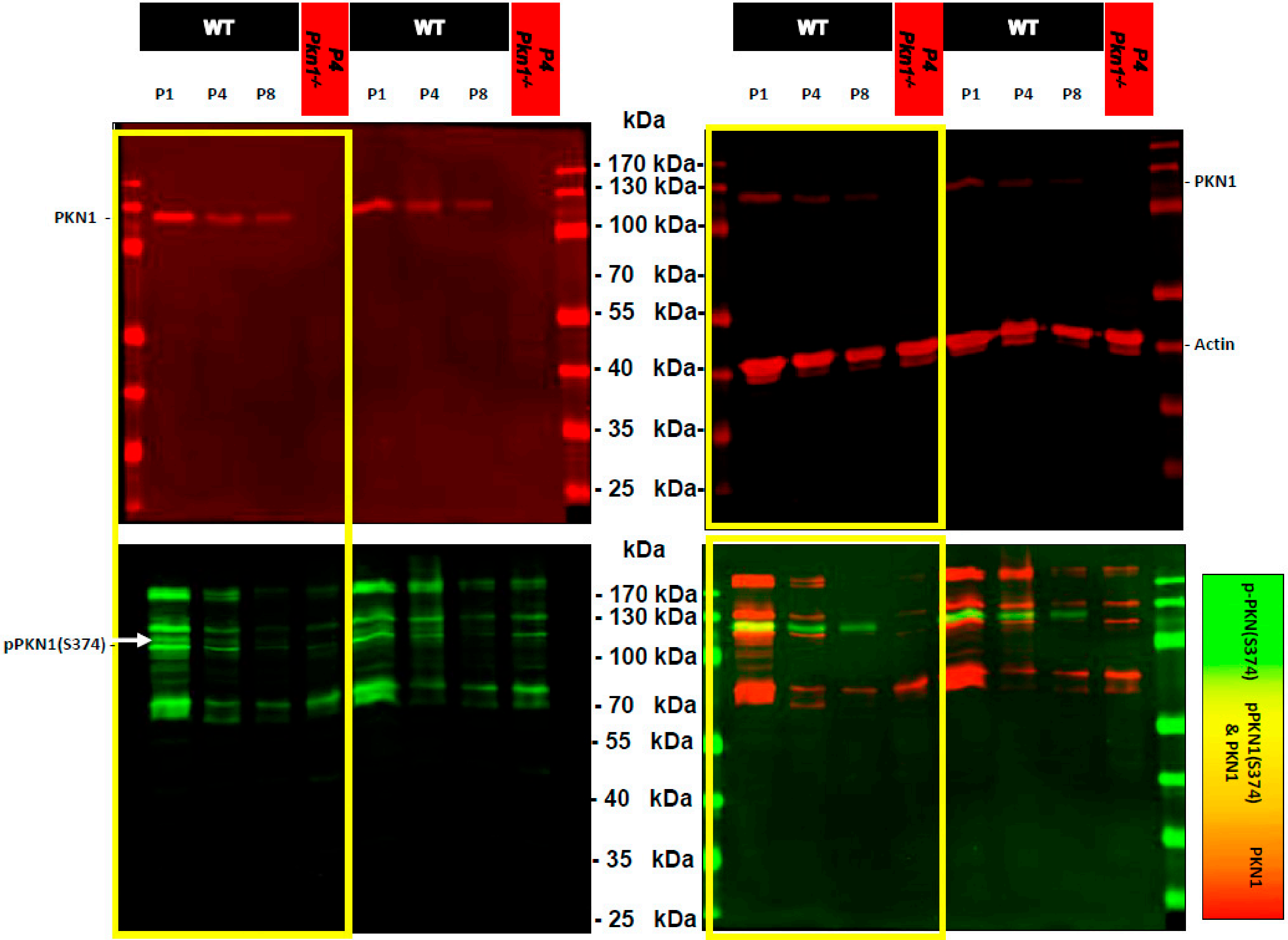

Figure S5. Uncropped blots from Figure 5b. Lanes taken for the show blot are shown in yellow.

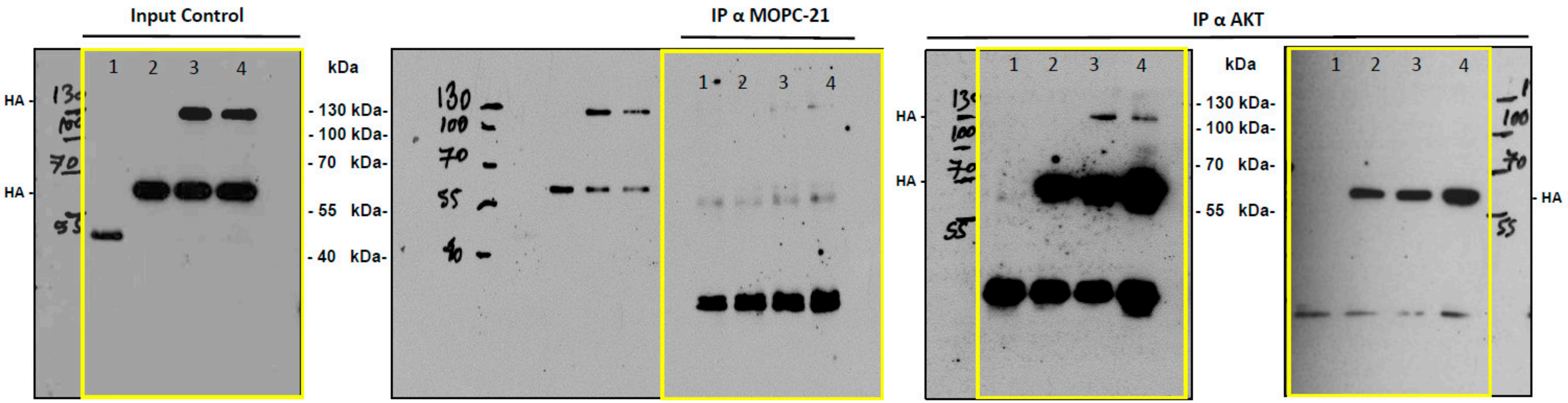

1 30 µg pEFneo
2 30 µg pEFneo AKT
3 15 µg pEFneo AKT + 15 µg *h*PKN1 wt
4 15 µg pEFneo AKT + 15 µg *h*PKN1 S374A

1 30 µg pEFneo
2 30 µg pEFneo AKT
3 15 µg pEFneo AKT + 15 µg *h*PKN1 wt
4 15 µg pEFneo AKT + 15 µg *h*PKN1 S374A

1 30 µg pEFneo
2 30 µg pEFneo AKT
3 15 µg pEFneo AKT + 15 µg *h*PKN1 wt
4 15 µg pEFneo AKT + 15 µg *h*PKN1 S374A

Figure S6. Uncropped blots from Figure 6a. Lanes taken for the show blot are shown in yellow.
